# SEE+ computerized classroom-based training enhances 7- to 10-year-olds' socio-emotional cognition through observation and inference

**DOI:** 10.1371/journal.pone.0330934

**Published:** 2025-09-02

**Authors:** Sveta Mayer, Iroise Dumontheil, Hannah R. Wilkinson, Kaska Porayska-Pomsta, Emily K. Farran, Andrew K. Tolmie, Denis Mareschal, the UnLocke Team

**Affiliations:** 1 UCL Faculty of Education and Society, University College London, Camden, London, United Kingdom; 2 Centre for Educational Neuroscience, Birkbeck University of London, Camden, London, United Kingdom; 3 Department of Psychological Sciences, Centre for Brain and Cognitive Development, Birkbeck University of London, Camden, London, United Kingdom; 4 Melbourne School of Psychological Sciences, University of Melbourne, Parkville, Victoria, Australia; 5 School of Psychology, University of Surrey, Guildford, Surrey, United Kingdom; Father Muller Charitable Institutions, INDIA

## Abstract

Smooth social interactions rely on children’s abilities to decode others’ social signals, which includes what an individual may say or do, and their facial emotional expressions. Failure can lead to exclusion from social groups. Consequently, a number of social and emotional learning (SEL) training programmes have been developed, with some evidence of positive impacts on those skills themselves and on academic achievement. Here, we present and evaluate the efficacy of a novel classroom-based computerized learning activity, called *SEE+ (Socio-emotional engagement through observation),* in supporting and enhancing 7- to 10-year-olds’ emotion recognition and theory of mind judgements through observation and inference. SEE+ involved observing four virtual characters interacting within social scenarios and inferring their mental states. Participants were recruited from across diverse school settings (rural, urban) in England as part of a large-scale randomised controlled trial (*n* = 5585; 7.3–11.0 years old, *M* age = 9.1, *SD* = 1.0) resulting in a mix of socio-economic and ethnic backgrounds. Mixed model ANOVAs were used to compare performance on socio-emotional tasks between the SEE+ group, an active control group and a teaching-as-usual control group. SEE + was associated with the equivalent of 4–6 months improvement in performance on the socio-emotional assessment tasks. Children showed near transfer effects of the intervention (i.e., when characters present in the computerized learning activity were used in the assessment), while no strong evidence of far transfer effects (i.e., when photographs of unknown children were used in the assessment) was found. Limitations were the use of pen-and-paper assessments with a reduced number of trials, and a possible ceiling effect in the older children in the photograph condition. Our findings point to the relative plasticity of younger children’s socio-emotional cognition and underscore SEE+ as an easy-to-use cost-effective socio-emotional resource for teachers to embed SEL in the school curriculum.

## Introduction

Children attending primary school education are faced with the challenge of navigating social interactions with a large group of peers of various age, both in the classroom and the playground. Effective social interactions rely on our observation and ability to decode others’ social signals [1], which include what an individual may say or do, their facial emotional expressions and their body language. Combined with our knowledge of others’ desires and beliefs and the context of social interactions, our “theory of mind” (ToM), also referred to as mentalizing, enables us to infer others’ thoughts, feelings and intentions, and to predict what they are going to do and/or feel [2,3]. Without these socio-emotional cognitive skills, children misjudge social interactions and can either fail to understand the context in which a situation is evolving or to know what an appropriate set of actions might be for them to take. This can lead to embarrassment and exclusion from social groups, such as friendship groups in social settings or peer-groups in educational settings. Good development of socio-emotional cognitive skills during early and middle childhood is associated with better relationships with teachers, parents and siblings, and more prosocial behaviour with peers in childhood [4] and, predicts higher academic attainment [5–7], as well as better physical and mental health into adolescence and adulthood [8]. Indeed, UNESCO is increasingly seeing socio-emotional cognition as a foundational cornerstone of learning and education across global contexts [9]. However, much remains to be understood regarding the multiple factors that influence the emergence and expression of social skills over the course of development [10,11]. The goal of the present research was to develop and evaluate the impact of a school-based intervention aiming to foster the development of socio-emotional skills in childhood.

Childhood is a period of continued improvements in socio-emotional cognition, during which individuals navigate increasingly complex social interactions. Children’s own emotions [12] and understanding of their own mind [13] become more complex over the course of development. Their ability to infer the emotional and mental states of others, i.e., mentalizing/ToM, also improves with age and depends on a combination of socio-emotional domain specific skills and non-specific cognitive skills. Socio-emotional specific skills include recognizing emotion expression from face and body gestures, empathy, perspective taking, and sensitivity to social and cultural norms in social interactions [14,15]. Non-specific cognitive skills include working memory, which allows children to keep and manipulate information in their mind, or inhibitory control and cognitive flexibility, which allow children to inhibit their own perspective or mental states when considering others’ perspective and mental states [10,11,16,17]. Research has shown that early and middle childhood are associated with improvements in the ability to make inference about others’ emotional and mental states from the face and eyes [18,19], gesture and body movement [20,21] as well as in complex situations, such as involving faux pas, irony or humour [22,23]. Children also become more flexible and accurate in inferring others’ perspectives, progress from understanding first-order false belief to second-order false belief (someone can hold a false belief about someone else’s belief) between 4 and 7–8 years of age (see [3,15,24–26] for reviews).

The neural systems supporting socio-emotional cognitive functions in complex social situations form the ‘social brain’, first postulated by Brothers and colleagues [27,28]. The social brain comprises a series of distributed, yet interrelated networks with multiple sensitive periods of postnatal plasticity and specialisation [29–31]. The processing of faces and facial expression in perceptual networks continues to mature in school age children, with evidence of increasing cortical specialisation over the course of development [32] and improvements in face processing ability up until age of 10–12 years old [33]. Neural processing of gestures and body movement – biological motion – also continues to specialize in 7- to 10-year-old children [20], and changes in brain activity continue to be observed in childhood and adolescence in action observation [34–36] and mentalizing networks during social cognition tasks [37,38].

Given that middle childhood is a sensitive period for the development of socio-emotional cognition and the positive impact of interventions on, e.g., children’s understanding of first and second order false belief and perspective taking, the utility of training socio-emotional cognitive skills in formal education has gained traction (see [39–42] for meta-analysis and reviews).

Social and emotional learning (SEL) curricula have been delivered in schools internationally to enhance children’s socio-emotional competence [43,44]. Overall, there is evidence of positive impacts of SEL curricula on socio-emotional competence [44–46] and on academic outcomes [46–48]. However, the variability, quality and value of SEL content and intervention resource provision remains highly variable [44,49,50]. In England, SEL is provisioned through the Personal, Social, Heath, and Economics (PSHE) curriculum themes: Health and Wellbeing, Relationships, Living in the Wider World and Economic Wellbeing and Careers. However, there is no specific theme for SEL within PSHE and no formal teacher training; schools are expected to tailor their local programme to reflect the needs of their pupils with guidance and resources available through the Department of Education, England and the PSHE Association [51,52]. We therefore developed and evaluated the efficacy of a novel classroom-based computerized learning activity focusing on emotion recognition and ToM, which could provide the basis for more consistent SEL provision within PSHE.

A challenge for SEL provision relates to the immersive, experiential and situated nature of social and emotional cognition, the learning of which may require new forms of pedagogies and learning supports [53]. Digital media and technologies are examples of novel ways to support the delivery of SEL curricula. In particular, a variety of digitally-enhanced environments involving social interaction between virtual characters have been explored with diverse learners, training social attention [54,55], emotional understanding [56–58] and social competencies [59], demonstrating the potential of such environments in a variety of learning contexts to support children’s development of social and emotional skills. Although resources of this kind are typically conceived of in terms of individual delivery, whole-class delivery may actually be more efficient, in reaching more children, and more effective, in allowing productive peer discussion that both elaborates and consolidates learning [60,61].

Recent evidence linking affective neuroscience with learning and development highlights the primary role of observing and engaging in social interactions in facilitating and supporting social and emotional cognition, mental health and academic motivation and engagement [62–64]. In the current study, we designed the Social and Emotional Engagement through Observation (SEE+) training as a computerized classroom-based tool delivered by classroom educators, which trained children to infer and reflect on the emotional and mental states of four virtual characters observed while they interacted with each other within complex social scenarios. The SEE+ learning activities delivered components of the English PSHE curriculum (see [65]) through learning activities in which children observed the characters interacting in social dilemmas or conflict. As a class, children reflected upon what the characters were doing, how they were feeling, and what they were thinking, and then collaboratively reached a consensus and answered questions, with feedback, about the characters’ action, thoughts and feelings and predicted how the characters may resolve the social situation they were in [61].

SEE + was delivered as part of a randomized control trial (RCT) [66–68]. The outcome measures consisted of pen-and-paper tasks assessing emotion recognition, emotional awareness and ToM skills of the children involved in the RCT. We also explored the extent to which any performance gains transferred to novel scenarios using the SEE+ characters in the training (near transfer) as well as images of real-life children (far transfer). We further compared the impact of SEE+ between 7– to 8-year-olds and 9– to 10-year-olds, who are at different points of the trajectory of socio-emotional development.

In summary, this research addressed the need to develop and validate an intervention that could foster socio-emotional skills during middle childhood, a key period of social cognitive development.

## Materials and methods

### The randomized controlled trial

SEE + was administered as part of a large-scale RCT involving 89 state-funded primary schools across England. The purpose of that trial was to evaluate the impact on mathematics and science achievement of a 10-week computerized learning activity, called Strop & Think, designed to help Year 3 (7–8 years old) and Year 5 pupils (9–10 years old) inhibit prepotent incorrect rapid responses when solving mathematics and science problems (see [66] and [68] for more details). Deeply held naïve beliefs and misconceptions often come to mind first and need to be inhibited to allow more reflective and accurate answers to come to the fore. In the RCT, the SEE+ learning activity was an active control for children in the Stop & Think (S&T) intervention condition. Fifty percent of children who took part were allocated to the Stop & Think intervention, 25% were allocated to SEE+ and 25% were allocated to a teaching as usual (TAU) condition. Randomization of classes to each condition was implemented by an independent external evaluator [67]. We leveraged this trial to assess concurrently the impact of the SEE+ learning activity on children’s socio-emotional cognition. After the completion of the mathematics and science achievement tests by the independent evaluators, our team assessed children who had taken part in the trial using a purpose designed pen-and-paper test of socio-emotional cognition, as well as a test of inhibitory control. Performance on the socio-emotional cognition tasks was not part of the official Stop & Think evaluation and is not reported on anywhere else. SEE + was delivered as part of the schools’ regular delivery of their PSHE curriculum. The study procedures were approved by the Research Ethics Committee, Department of Psychological Sciences, Birkbeck College, University of London. Ethics approval number: 161741. The ethics approval date was 20/03/2017 and recruitment was between 01/04/2017 and 25/07/2018. Written informed consent was obtained from the Head of School. Minors were involved and parents or guardians were informed via written correspondence and provided with the opportunity to give opt-out informed consent from the testing associated with the evaluation of SEE + .

### Participants

Participants were recruited from state-funded, non-fee-paying primary schools in England. Of the 6,486 pupils recruited to the main trial, we failed to collect data for 1,060 pupils (16%). This is comparable to the rate of 10% of persistent absentees in English primary schools [69]. A second wave of intervention and data collection was carried out in a smaller number of schools around London (see [66] for details) and provided 194 additional participants, resulting in a final sample of 5,620 children for the current study. Missing data was evenly distributed across most schools (87 schools had some missing data) and Year groups (49% from Year 3 and 51% from Year 5). The proportion of missing data by intervention condition (SEE + 34%, S&T 41%, TAU 25%) did not differ from the proportion of included data by intervention condition (SEE + 27%, S&T, 50%, TAU, 23%, Χ^2^(2) = 3.61, *p* = .165). The mean school percentage of pupils who had ever been eligible for Free School Meals (FSMs; which can be used as a proxy for socio-economic status; data from 2017/2018 Spring school census [67]), was 16.2% (*SD* = 12.4), which is comparable to the 13.7% of pupils eligible for FSM in primary schools in January 2018 [70]. School percentage of FSM was not available for 301 pupils (4.9% of pupils with socio-emotional data). Thirty-five children were excluded from the analyses because of poor task performance (see below), resulting in a total analysed sample of 5,585 children (Table 1). Age information was missing for 4.5% of children. For the 5,346 children with age data, there was no difference in mean age between the three conditions in Year 3 (*F*(1,2631) = 1.12, *p* = .328, η_p_^2 ^= .001) or Year 5 (*F*(2,2709)=0.24, *p* = .789, η_p_^2^ = .001) (Table 1). The exact gender distribution was not available for our analyses because of the anonymization of the full-scale Stop & Think RCT data following the end of the trial; however, the RCT evaluation team reported no imbalance in gender by intervention condition or by age group, and no significant effects related to gender in the Stop & Think RCT [67]. In line with the English government’s guidelines for participation in the national end of primary Standardised Assessment Tests (SATs), all children present on the day of the test were invited to take part in the study; there were no exclusion criteria. This included some children with special educational needs or other support plans who normally participated in the class.

**Table 1:** Age distribution of participants.

Year group	Condition	*n*	Age (years)		
			*n* missing	*M*	*SD*
Year 3	Teaching as usual	631	52	8.08	0.30
	Stop & Think	1299	3	8.07	0.29
	SEE+	808	49	8.06	0.29
	Total	2738	104	8.07	0.29
Year 5	Teaching as usual	641	4	10.07	0.31
	Stop & Think	1485	131	10.06	0.29
	SEE+	721	0	10.06	0.30
	Total	2847	135	10.06	0.30

### Schools, training, and testing conditions

Details of the school recruitment procedures, geographic distribution and other school characteristics can be found in the Stop & Think RCT final report [67]. Briefly, however, the schools were recruited by self-nomination from throughout England. They were all state-funded primary schools with a mix of urban and rural settings and a mix of small (mixed class) and multiform (multiple classes per year group) entry schools. No financial incentives to take part were provided. The computerized training sessions (described below) were led by the regular class teacher or teaching assistant as part of normal classroom activities. Post-intervention testing (described in more detail below) took place as a whole group activity within the children’s normal classrooms and was led by a research assistant not involved in providing any operational support for the school during the Stop & Think RCT (i.e., blind to the class’s RCT condition allocation).

### SEE+ intervention

The SEE+ computerized, classroom-based learning activity was designed to develop socio-emotional cognition through the *observation* of four virtual SEE+ characters’ interactions in social scenarios involving social dilemmas or conflict and the *inference* of the characters’ mental states, e.g., intentions, beliefs, psychological dispositions and feelings. The social interactions between the characters, two girls and two boys aged 6–10 years of White or Black African ethnicity were animated (see S1 File Appendix).

SEE+ learning activities were completed as a whole class and animations were projected onto a screen or white board, with the teacher leading the class through the activities and eliciting responses from the class either through majority consensus or selecting a different child each time to provide an answer. Answers were entered into the computerized interface. Each activity lasted 12 minutes and could be used as a springboard for further discussion if desired. Teachers were asked to complete the 30 successive activities over the course of 10 weeks, three times a week, during PSHE lesson time. The activities were designed so that the task structure and duration matched as closely as possible that of the tasks presented in the other RCT training condition, Stop & Think, while not requiring any mathematics or science reasoning. In this way, SEE+ and Stop & Think could both act as active control activities for each other [66–68].

Thirty social scenarios involving the four SEE+ virtual characters in social dilemmas or conflict were created, informed by the English PSHE curriculum [65] and Social Communication, Emotion Regulation and Translational Support (SCERTS) intervention and assessment programme [71], and rendered into animations. Eight archetypical activities were developed, each representing a different aspect of social-emotional cognition, and each derived from standard socio-emotional paradigms used in the developmental psychology literature focusing on autism research (Table 2); the eight activities were then presented in increasing order of complexity. The scenarios were created to represent complex social interactions that might be observed in real-life by children aged 7–9 years.

**Table 2:** List of the archetypical activities undertaken by children within the SEE+ computerized learning activities with primary socio-emotional cognitive target domains.

Order of progression	SEE+ Primary Domain Targeted in Archetypical Learning Activities	References
1	Face processing skills with respect to recognizing facial emotion expressions (derived from emotion recognition task)	[72]
2	Inferring psychological disposition from pictures of the eye regions (derived from the Reading the Mind in the Eyes task)	[18,73]
3	Inferring psychological disposition from body motion cues (derived from action observation task)	[74,20]
4	Sensitivity to complex social interactions (derived from the Faux Pas task)	[22]
5	Empathy with respect to social exclusion (derived from the Cyberball task)	[75,76]
6	Perspective taking, theory of mind with respect to others’ mental states (derived from the Director task)	[77,78]
7	Perspective taking, theory of mind with respect to other persons’ intentions (derived from the Sandbox task)	[79]
8	Social perspective taking, theory of mind with respect to others’ beliefs and intentions within social situations (derived from the Strange Stories task)	[23,80]

**Fig 1:**
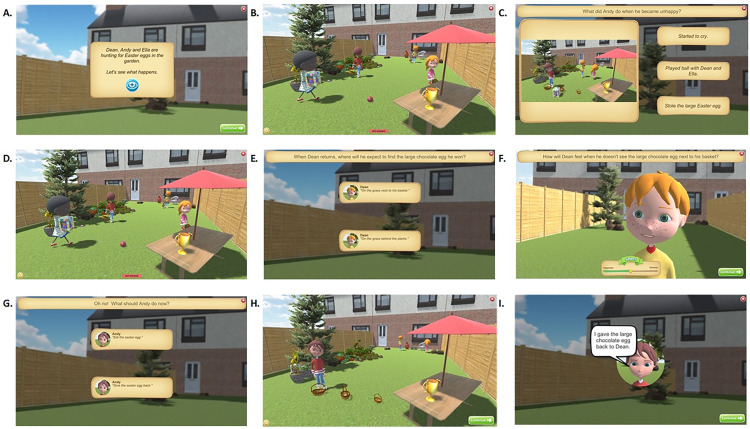
Structure of a SEE+ Learning Activity. In phase 1 (A-C), the context was first provided (A) and children were prompted to watch the first animation (B). Children were then asked what the characters were doing with multiple choice questions (C) followed by feedback. In phase 2, (D-F), children watched a replay of excerpts of the animation (D) and were asked to infer a character’s mental state (E) and for example rate the intensity of their social emotions (F). In phase 3 (G), children were asked to predict what the characters might do to resolve the social conflict. In phase 4 (H-I), the children were given feedback by watching a second animation showing the characters resolving the situation (see S1 File Appendix for a description of this scenario).

The virtual characters were endowed with a rich set of facial expressions (see S1 File **Appendix**) and arm and hand gestures, allowing the depiction of complex social emotions such as intimidation, ostracism, embarrassment, envy, empathy, guilt, shame, and joy [59,81,82]. The virtual characters’ interactions were scripted as sequenced chains of characters’ social and emotional: (a) actions, reactions and counteractions given the social dilemma or conflict up to the point of a cliff-hanger followed by (b) counter-reactions with each other to resolve the social situation to depict characters’ multiple mental states [26,83–85]. Learning activities were structured as four phases with two episodes of observation interleaved with reflective questions and feedback with a narrator’s voice over recording guiding the learner through the phases (see Fig 1 and S1 File Appendix for more details).

### Measures

Socio-emotional cognition was assessed post-training using five custom designed booklets which were administered to individuals within whole class settings. The first four booklet tasks, adapted from the NEPSY II nonverbal subsets for the Social Perception domain affect recognition and theory of mind contextual task measures normed for 5- to 16 year old children [86,87], required judgements of either other’s emotions, i.e., emotion recognition (ER), or a mental state judgement involving ToM, with either photographs of real face (to assess far transfer) or cartoon face stimuli (to assess near transfer) similar to those used in the SEE+ and S&T interventions. Bespoke booklets enabled us to establish internal validity for our outcome measure for transfer effects of SEE+ intervention.

**Fig 2:**
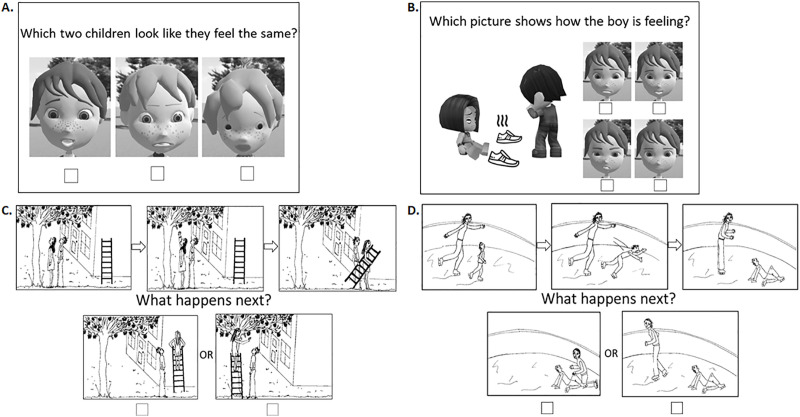
Example items from the socio-emotional tasks booklets. Children were presented with: (A) three (minimum) to six (maximum) photograph images of real faces (not shown) or SEE+ character faces, and asked to identify which two children look like they felt the same and, (B) a social situation involving two protagonists interacting with an object and then asked to infer what the child whose face they could not see would feel based on the social situation, choosing between four options with real faces (not shown) or SEE+ character faces. SEE+ faces were similar to those used in the SEE+ and Stop & Think computerized activities. Children were then showed cartoon vignettes illustrating physical causality (not shown), cognitive theory of mind (C) or affective theory of mind (D) scenarios and asked to infer what happens next.

The booklets were completed in a fixed order (real faces ER, cartoon faces ER, real faces ToM, cartoon faces ToM) to reflect increasing complexity in socio-emotional judgement; each included one practice item and seven test items. The ER task presented children with whole face stimuli of real children or SEE+ characters and asked them to identify which two children out of three (3 items), four (2 items) or six (2 items), as proxy of increasing social groups, looked like they felt the same (Fig 2A). The ToM tasks presented children with seven social situations (7 items) involving two protagonist interacting with an inanimate object where one of the protagonist’s face is not seen; each item included a set of four face image stimuli of photographs of real children’s faces or SEE+ character faces and children were asked to infer which of these four faces represented what the protagonist whose face they could not see felt in that social situation (Fig 2B). Scores on each booklet ranged from 0 to 7.

The task stimuli used for the four booklets were whole face stimuli of SEE+ character faces created using facial expression blending developed during the SHARE-IT project [59] and real faces of photographs of 7–10 year-old children with varied gender and ethnicity from the JimStim database developed by Jim Tanaka, University of Victoria, British Columbia, Canada. The socio-affective scenarios of dyad character interaction face expression and body gestures were created by the authors using the My School Avatar App 3D Avatar Creator developed by BuddyPoke LLC [88] with images of object and wavy line icons created by Freepix: Flaticon.com [89] (Fig 2B).

The fifth booklet presented a ToM vignette task adapted from Sebastian et al. [90] to measure children’s first level ToM. On each page, children were shown three images at the top illustrating a story and were asked to choose “What happens next?” between two further images presented at the bottom of the page representing two choices of ending (Fig 2C-D). There was one training item (cognitive ToM) followed by seven test items, which included one physical causality, two cognitive ToM (e.g., Fig 2C) and four affective ToM scenarios (e.g., Fig 2D). Each vignette scenario portrayed two characters. Affective ToM cartoons required children to infer how one of the characters in the story would react to their companion’s affective state in order to choose the correct ending. Cognitive ToM vignettes required children to make an inference based on the intentions of the story characters. The physical causality item required an understanding of cause and effect. Items were presented in a fixed order across participants. Score on this booklet ranged from 0 to 6; the physical causality item was not included in the analyses.

### Procedure

A researcher visited each school at the beginning of the project to install the software on the classroom computer and provide training to teachers. Over the course of the intervention the researcher kept in touch with the school regularly and was able to answer queries from teachers.

Administration of post-training assessments were carried out by a different researcher who was blind to the trial condition that the class had been allocated to. The post-intervention evaluations were carried out in a whole class format. After having introduced the five booklets the researcher handed out pens and the booklets to the children in the class. The researcher explained the booklets were going to be completed one at a time. For each booklet the researcher first showed the practice item and explained the task to the children. No feedback was provided, and, when the children had completed the practice, they were given 2 min to answer all questions in the booklet. When the time was up the researcher said “stop” and children had to put their pens down on their desks.

### Data analysis

All data were analyzed using SPSS version 29. Graphs were prepared using RStudio 2024.04.1 and Excel. Means and standard deviations of total scores for the first four task booklets measuring performance on socio-emotional cognition were calculated for Year 3 and Year 5 children. Outliers were defined as children scoring above or below 3.29 SDs from the mean (for their Year group). Sixteen Year 3 participants (2 SEE + , 11 Stop & Think, 3 TAU) and 19 Year 5 participants (6 SEE + , 10 S&T, 3 TAU) had performance below 3.29 SDs from the mean and were removed from all analyses.

For our main analyses, we investigated whether socio-emotional task scores were significantly different between children who had participated in SEE+ and children in the S&T and TAU control groups. We conducted a 3 (Condition: SEE + ; S&T; TAU) x 2 (Task: ER; ToM) x 2 (Face type: Cartoon; Real) x 2 (Year group: Year 3; Year 5) mixed model repeated measures ANOVA, with intervention condition and Year group as between-subject factors and task and face type as within-subject factors. This allowed us to test for an effect of the intervention, and to examine whether the effect may be modulated by Year group, the type of task, and the type of stimuli, contrasting items using SEE+ faces (similar to stimuli used in the SEE+ and S&T interventions) to items using photographic images of real faces (not used in the interventions). Here ‘Cartoon’ represents SEE+ character face stimuli used in SEE+ training and ‘Real’ represents photographs of children’s faces not used in the training.

Performance on the cognitive and affective ToM task of the fifth booklet was analyzed separately using a 2 (Condition: SEE + ; Stop & Think; TAU) x 2 (Year group: Year 3; Year 5) ANOVA. Significant main effects and interactions were followed-up with Bonferroni corrected post-hoc pairwise comparisons.

Pupils within the same school may share characteristics of that school’s context (e.g., geographical location, school percentage of pupils with free school meals; school resources; number of pupils in the school; teacher/child ratio; curriculum approach) but pupils across different schools may differ in these school-level characteristics. Similarly, pupils within the same class share characteristics in, e.g., peer interactions and teacher style. This violates the assumption of a single-level linear model that data are independent. We therefore repeated the analyses using multilevel linear mixed models, with pupils nested within classes nested within schools. There were 88 schools, with an average of 63.5 participants per school (range 4–171) and 260 classes, with an average of 21.5 participants per class (range 2–58). The *lmer* function of the lmerTest R package was used to run the analyses, the *anova* function to provide F-test statistics, the *fixef* function of the lme4 package to estimate fixed effects, and the emmeans package to obtain estimated means. Data from booklets 1–4 were analysed entering task (ER, ToM), face type (cartoon, real), year (Year 3, Year 5) and condition (SEE + , TAU, S&T) as fixed effects and participants nested within classes nested with schools as random effects, with the equation: score ~ task * face_type * year * condition + (1 | school/class/participant), estimating random intercepts for participants, classes and schools. For the cognitive and affective ToM task of the fifth booklet there was no within-subject task factor, and the equation was: score ~ year * condition + (1 | school/class). Models were estimated using the Restricted Maximum Likelihood (REML) method. We note that a comparison (using the *anova* function) of the models with class and school as random effects was not better than a model with school only, while it was better than a model with class only, but we used the full nested model to capture the full nested structure of the data.

Data files for this study can be found on the Open Science Framework (https://osf.io/vesku/?view_only=abfe9501daa84feaaa57b0ca16486e4e).

## Results

A mixed model repeated measures ANOVA was run with task (ER; ToM) and face type (cartoon; real) as within-subject factors and Year (Year 3; Year 5) and training condition (SEE + ; Stop & Think; TAU) as between-subject factors. Descriptive statistics of the scores are presented in Table 3.

**Table 3:** Descriptive statistics (M (SD)) of scores on the five socio-emotional cognition task booklets.

		SEE+^a^	S&T^b^	TAU^c^
**Emotion recognition – Cartoon faces** (range 0–7)	**Year 3**	4.08 (1.22)	3.84 (1.24)	3.81 (1.19)
	**Year 5**	4.38 (1.16)	4.22 (1.21)	4.09 (1.16)
**Emotion recognition – Real faces** (range 0–7)	**Year 3**	3.94 (1.5)	3.79 (1.52)	3.90 (1.46)
	**Year 5**	4.56 (1.49)	4.61 (1.40)	4.52 (1.42)
**ToM**^**d**^ **- Cartoon faces** (range 0–7)	**Year 3**	3.71 (1.35)	3.61 (1.32)	3.61 (1.36)
	**Year 5**	4.27 (1.30)	4.12 (1.29)	4.19 (1.30)
**ToM – Real faces** (range 0–7)	**Year 3**	4.40 (1.42)	4.24 (1.45)	4.39 (1.41)
	**Year 5**	4.91 (1.27)	4.90 (1.27)	4.81 (1.26)
**Cognitive and affective ToM** (range 0–6)	**Year 3**	3.25 (0.94)	3.26 (0.99)	3.17 (1.03)
	**Year 5**	3.33 (0.92)	3.39 (0.87)	3.29 (0.91)

^a^SEE+ = Social and Emotional Engagement through Observation; ^b^S&T = Stop & Think; ^c^TAU = Teaching as usual; ^d^ToM = Theory of mind.

Sample sizes: Year 3: SEE + **n* *= 808, S&T **n* *= 1299, TAU *n* = 631; Year 5: SEE + **n* *= 721, S&T **n* *= 1485, TAU *n* = 641.

Statistical results of the mixed model ANOVA are presented in Table 4. Analyses were repeated using a multilevel linear mixed model with participants nested within classes nested within schools. Statistical results of this model are also presented in Table 4. The results of these analyses were broadly similar with the mixed model ANOVA results. Only significant effects of the training condition factor of interest are discussed further below.

**Table 4:** Statistical results of the Task (emotional regulation; theory of mind) x Face type (cartoon; real) x Year (Year 3; Year 5) x training Condition (SEE+; Stop & think; teaching as usual) mixed model repeated measures ANOVA and multilevel linear mixed model analyses performed on the socio-emotional cognition booklets scores.

Effect	Mixed ANOVA results				Multilevel linear mixed model results			
	*df*	*F*	*p*	η_p_^2^	*df*	*F*	*p*	Estimates of effects
Task	1, 5579	37.64	<.001	.007	1, 16737	46.97	<.001	−0.38
Face type	1, 5579	686.60	<.001	.110	1, 16737	610.33	<.001	−0.14
Year	1, 5579	478.22	<.001	.079	1, 218	352.24	<.001	0.30
**Training condition**	**2, 5579**	**10.55**	**<.001**	**.004**	**2, 180**	**4.90**	**.008**	**TAU: −0.26, S&T: −0.20**
Task x Face type	1, 5579	290.37	<.001	.049	1, 16737	250.71	<.001	0.83
Task x Year	1, 5579	1.14	.285	<.001	1, 16737	1.43	.233	0.27
Task x Training condition	2, 5579	1.80	.165	<.001	2, 16737	2.25	.106	TAU: 0.17, S&T: 0.14
Face type x Year	1, 5579	28.37	<.001	.005	1, 16737	25.22	<.001	0.32
**Face type x Training condition**	**2, 5579**	**5.75**	**.003**	**.002**	**2, 16737**	**5.11**	**.006**	**TAU: 0.23, S&T: 0.09**
Training condition x Year	2, 5579	2.73	.065	.001	2, 117	0.13	.882	TAU: 0.04, S&T: 0.04
Task x Face type x Year	1, 5579	38.69	.001	.007	1, 16737	33.40	<.001	−0.38
Year 3 Face type	1, 2735	213.05	<.001	.072	1, 8205	186.06	<.001	−0.14
Task x Face type	1, 2735	260.32	<.001	.087	1, 8205	224.34	<.001	0.83
Year 5 Face type	1, 2844	508.25	<.001	.152	1, 8532	460.11	<.001	0.19
Task x Face type	1, 2844	60.85	<.001	.021	1, 8532	52.64	<.001	0.45
Task x Face type x Training condition	2, 5579	2.65	.071	<.001	2, 16737	2.28	.102	TAU: −0.13, S&T: −0.15
Task x Year x Training condition	2, 5579	0.67	.512	<.001	2, 16737	0.84	.433	TAU: 0.03, S&T: −0.14
**Face type x Training condition x Year**	**2, 5579**	**4.48**	**.011**	**.002**	**2, 16737**	**3.98**	**.019**	**TAU: 0.01, S&T: 0.11**
Cartoon faces **Training condition**	**2, 5579**	**18.89**	**<.001**	**.007**	**2, 208**	**11.35**	**<.001**	**TAU: −0.26, S&T: −0.23**
Training condition x Year	2, 5579	0.05	.953	<.001	2, 137	<0.01	1.000	TAU: −0.02, S&T: 0.07
Real faces Training condition	2, 5579	1.96	.141	<.001	2, 177	1.02	.362	TAU: −0.05, S&T: −0.09
**Training condition x Year**	**2, 5579**	**5.60**	**.004**	**.002**	2, 173	0.41	.663	TAU: 0.10, S&T: 0.13
Task x Face type x Training condition x Year	2, 5579	0.98	.375	<.001	2, 16737	0.85	.429	TAU: −0.12, S&T: 0.09

Significant effects involving the training condition factor of interest are highlighted in bold. TAU: teaching as usual; S&T: Stop and Think.

**Fig 3:**
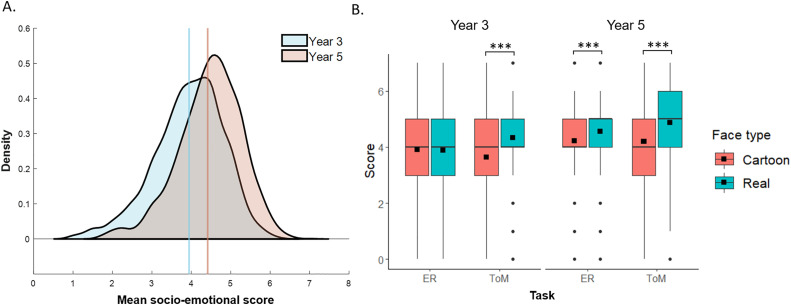
Socio-emotional scores as a function of task, face type and year group. (A) Density plot as a function of Year; estimated means are indicated by the vertical lines. (B) Boxplot illustrating the interaction between task (emotion recognition (ER), theory of mind (ToM)), face type and Year, means are indicated by the black squares. *** *p* < .001.

Children were less accurate in the emotional recognition (*M* correct answers (maximum score 7) = 4.146, *SE* = 0.015) than the ToM tasks (*M* = 4.262, *SE* = 0.015), and more accurate for real (*M* = 4.414, *SE* = 0.015) than cartoon faces (*M* = 3.994, *SE* = 0.013). In addition, children in Year 5 (*M* = 4.414, *SE* = 0.015) were more accurate than children in Year 3 (*M* = 3.943, *SE* = 0.017) (Fig 3A). These main effects were modulated by two-way interactions between task and face type and between face type and Year, as well as a three-way interaction between task, face type and Year (Table 4).

Follow-up of these interactions indicated that first, while children were more accurate for real faces than cartoon faces in both ER and ToM tasks (*p*’s < .001), the difference was larger for ToM (*M*_diff_ = 0.688, η_p_^2 ^= .144) than ER (*M*_diff_ = 0.151, η_p_^2 ^= .008). Similarly, while both Year 3 and Year 5 children were more accurate for real faces than cartoon faces (*p*’s < .001), the difference was larger for Year 5 (*M*_diff_ = 0.505) than Year 3 children (*M*_diff_ = 0.334, Table 4). The three-way interaction reflected the fact that in both Year 3 and Year 5 the difference in accuracy between real and cartoon faces was greater in ToM than ER tasks, but this difference between tasks was smaller in Year 5, than in Year 3 (Table 4). In Year 5, higher accuracy for real faces than cartoon faces was observed both for ToM (*M*_diff_ = 0.675, *p* < .001, η_p_^2 ^= .145) and ER tasks (*M*_diff_ = 0.334, *p* < .001, η_p_^2 ^= .037), while in Year 3 the difference between face types was observed in ToM (*M*_diff_ = 0.701, *p* < .001, η_p_^2 ^= .144) but not ER tasks (*M*_diff_ = −0.033, *p* = .303, η_p_^2^ < .001) (Fig 3B).

**Fig 4:**
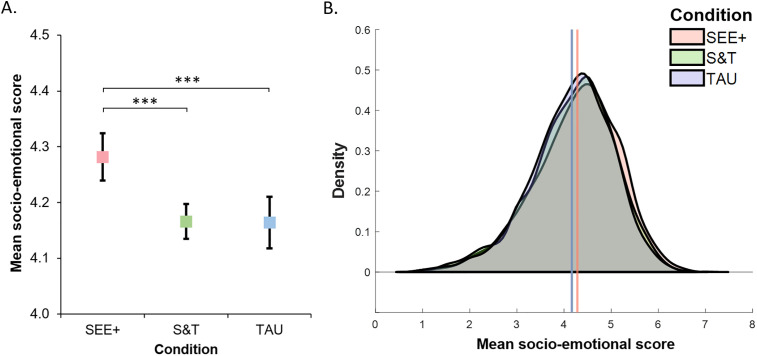
Total socio-emotional score as a function of training condition. (A) Mean and 95% confidence interval of the total score; (B) Density plot as a function of training condition; estimated means are indicated by the vertical lines – note the S&T and TAU means are overlapping. S&T = Stop & Think; TAU = Teaching as usual; *** *p* < .001.

The key effects of interest were those that involved the intervention condition factor. There was a main effect of training condition (Table 4). Follow-up Bonferroni corrected pairwise comparisons indicated that children in the SEE+ condition (*M* = 4.282, *SE* = 0.022) were more accurate than children in the Stop & Think (*M* = 4.166, *SE* = 0.016, *p *= .007) and TAU conditions (*M* = 4.164, *SE* = 0.024, **p* *= .004), while the two control groups did not differ (*p* = 1.0) (Fig 4). The difference in score between children in SEE+ and those in S&T/TAU (0.117), when compared with the difference between Year 3 and Year 5 children (0.471) suggests the intervention is associated with the equivalent of 6 months improvement in performance on the socio-emotional tasks. The multilevel linear mixed model analysis gave a slightly lower estimate of the equivalent of 4 months of improvement in performance in SEE + compared to S&T/TAU (estimated means: *M*_SEE+_* = *4.262, *SE* = 0.032; *M*_S&T_ = 4.165, *SE *= 0.025; *M*_TAU_ = 4.182, *SE *= 0.027; *M*_Year3_ = 3.938, *SE* = 0.026; *M*_Year5_ = 4.468, *SE = *0.027). For this analysis the ICCs were 0.019 for the class-level and 0.004 for the school-level.

**Fig 5:**
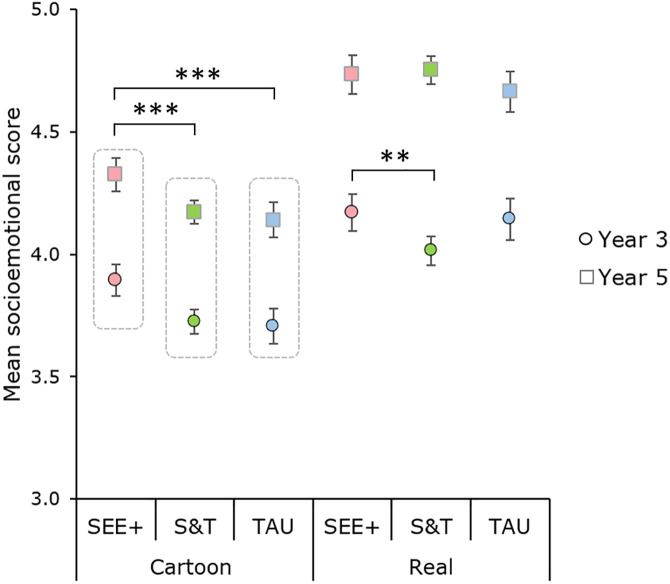
Estimated mean socio-emotional score (and 95% CI) as a function of face type, intervention condition and year group. There was a three-way interaction between these factors. Follow-up analyses indicated that for cartoon faces performance was better in the SEE+ condition than in the control conditions across Year groups, while for real faces children in the SEE+ condition only outperformed children in S&T, and only in Year 3. S&T = Stop & Think; TAU = Teaching as usual; ***p < .001; **p < .01.

This main effect was modulated by a two-way interaction between face type and training condition, which was further modulated by a three-way interaction between face type, training condition and Year (Table 4). Follow-up analyses indicated that for cartoon faces the improved performance observed in SEE + was similar in the two Year groups. For these faces, there was no interaction between Year and training condition (Table 4), with a significant main effect of training condition showing better performance in the SEE+ condition (*M* = 4.111 (*SE* = 0.024)) than the Stop & Think (*M* = 3.948 (*SE* = 0.018), *p *< .001) and TAU conditions (*M* = 3.923 (*SE* = 0.026), **p* *< .001), which did not differ from each other (*p* = .672, with Bonferroni correction). The pattern was different for real faces, where the SEE+ benefit was observed in Year 3 only. The main effect of training condition was not significant, but there was a significant interaction between Year and training condition (Table 4). Follow-up analyses in each Year group separately indicated that the main effect of intervention condition was significant in Year 3 (*F*(2, 2735) = 5.53, *p* = .004, η*_p_*^2 ^= .004) but not in Year 5 (*p* = .200, η*_p_*^2 ^= .001). In Year 3, Bonferroni corrected pairwise comparisons showed that children in SEE+ were more accurate than children in S&T (*p* = .007), but no other comparison reached significance (*p*’s > .059) (Fig 5). The multilevel linear mixed model analysis also showed a significant interaction between face type and training condition, led by higher scores in SEE+ than S&T and TAU conditions (*p*’s < .001) for cartoon faces, but no effect of training condition for real faces. However, while the three-way Face type x Condition x Year group interaction was also significant, the follow-up analyses indicated that the training condition by Year group interaction was significant in neither Year 3 nor Year 5, suggesting the observed specific difference between S&T and SEE+ for real faces in Year 3 may have been driven by class or school contexts.

Finally, scores on the affective and cognitive ToM vignette task (fifth booklet) were analyzed using an ANOVA, entering training condition (SEE + ; Stop & Think; TAU) and Year group (Year 3; Year 5) as between subjects fixed factors (see Table 3 for descriptive statistics). There was a main effect of Year group (*F*(1, 5579) = 16.26, *p* < .001, η*_p_*^2 ^= .003), whereby Year 5 children (*M* = 3.334 (*SE* = 0.019)) outperformed Year 3 children (*M* = 3.227 (*SE* = 0.019)). There was also a main effect of Condition, (*F*(2, 5579) = 4.19, *p* = .015, η*_p_*^2 ^= .001), however, contrary to predictions, post-hoc Bonferroni corrected pairwise comparisons indicated that the SEE+ (*M* = 3.289 (*SE* = 0.024)) group did not differ from the control groups, but the S&T group (*M* = 3.322 (*SE* = 0.018)) outperformed the TAU group (*M* = 3.231 (*SE* = 0.026), *p* = .008). The Year x Condition interaction was not significant (*F*(2, 5579) = 0.44, *p* = .64, η*_p_*^2 ^< .001). This analysis was repeated using a multilinear mixed models including clustering by classes nested within schools. While the main effect of Year group remained significant (*F*(1, 190)= 10.24, *p* = .002, estimate Year 5 vs. Year 3 = 0.066), and the Year x Condition interaction remained not significant (*F*(2, 120) = 0.43, *p *= .65), the main effect of Condition became at trend level (*F*(2, 192) = 2.88, *p* = .059, effect estimates: S&T vs. SEE+ = 0.003, TAU vs. SEE+ = −0.073).

## Discussion

Making appropriate socio-emotional judgements is critical to children’s social decision making. Primary school, in which children are confronted with large groups of similar aged peers, is a common context for these decisions. We developed a computerized, classroom-based, learning activity called SEE+ designed to improve socio-emotional decision making in 7–10-year-olds through vicarious learning. The intervention provided elements of age appropriate PSHE curriculum. It was delivered by the teacher through whole class activities and could be used as a springboard for further activities and discussions as part of normal lessons.

### Performance on the socio-emotional cognition booklets

Analysis of the socio-emotional cognition tasks indicated that children were more accurate in the ToM than the emotion recognition task, and more accurate for real faces than cartoon faces. As the cartoon faces used are specific to the intervention and not derived from, e.g., a well-known television cartoon, the children will have had much more exposure to and experience of real children’s emotional expressions, which likely explain the better performance observed for this type of stimuli. This interpretation fits with the fact that the older children showed a greater difference between the real and cartoon faces. It was expected that children would perform better on the simpler emotion recognition tasks than the ToM tasks, as they are more complex and require understanding a social context, however it is possible that the social context gave additional cues to the children and helped them choose the appropriate emotion. Note that the difference in score between these tasks was small. As expected, considering the prolonged maturation of socio-emotional cognition [4,25,33], 9–10-year-olds (Year 5) children showed better performance than the 7–8-year-olds (Year 3). Our findings also reproduce the age effects validating NEPSY II measures for 5- to 16-year-old children found by Korkman et al., [91].

### Effects of the SEE+ intervention

We found that children who used the SEE+ activities for 10 weeks, three times a week, showed better performance on a pen-and-paper socio-emotional task than children in control groups. This suggests that the SEE+ activity improved the children’s ability to recognise emotions from facial expression and body postures and to infer others’ emotions based on a social scenario. Both Year 3 and Year 5 children showed near transfer effects to SEE+ cartoon character faces. The mixed ANOVA suggested far transfer to real faces which had not been used in the intervention in Year 3 children. This may reflect the fact that the younger children’s brain and socio-emotional skills may be more rapidly developing at this age, leading to greater learning and transfer. An understanding of possible developmental windows of enhanced learning of cognitive skills is emerging, with potential for informing education policy in schools ( [31,92,93]). However, this finding is mitigated by the lack of difference between SEE+ and the TAU control group, a possible ceiling effect, as the older children’s showed relatively high performance in all three intervention conditions when probed with photographs of other children, and the lack of similar effect in the multilevel linear mixed models, suggesting the effect may have been inflated by school- or class-level contexts [94]. Lack of far transfer is a general issue in cognitive training interventions (e.g., [95,96]). Similar improvements were observed whether children were asked to focus only on emotional facial expressions, in the emotion recognition task, or to also consider the character’s mental states, in the ToM task. In contrast, no benefits of SEE+ were observed in the cognitive and affective ToM vignette task.

A meta-analysis of 32 articles of ToM training interventions found that ToM skills could be trained, with a combined moderate effect size (**g* *= 0.75, [39]). Larger effects were observed for studies with longer sessions, and those with more sessions, but the effect of ToM training was reduced when the overall training intervention duration was longer. Mean age of the samples in the studies reviewed ranged from 3.4 to 13.7 years and was not found to moderate the impact of ToM training. However, the studies with a mean age above 7 years old all included atypical populations (hearing impaired or with ASD). Our findings therefore extend this previous research to older typically developing children, demonstrating that ToM skills can be fostered in 7- to 8-year-olds. A recent meta-analysis of 424 SEL intervention studies from 53 countries found a small beneficial impact on SEL skills (*g* = 0.22, [44]) and no moderating effect of the samples’ mean age, however there was a potential for publication bias for SEL skills outcomes. The present study’s work is at the intersection of this literature, with specific cognitive measures of emotion recognition and ToM, and a universal SEL intervention. Further work will be needed to extend and replicate the findings of potentially greater plasticity of socio-emotional cognitive skills in 7–8 than 9–10-year-old typically developing children.

### Strengths and limitations

The results of this study suggest that SEE + is an effective and economical way of delivering SEL as part of the PSHE curriculum provision. Our findings suggest that children’s socio-emotional cognition is relatively plastic and that even a short burst of training can help improve socio-emotional cognitive skills. The success of this programme also opens the door to simple cost-effective training for children with greater socio-emotional difficulties. SEE + was administered as a whole-class, universal intervention. Our current sample consisted of a large natural sample of children in state funded schools across different regions of England, which means the results are not limited to a specific subset of the population. It is possible that some children with social-emotional difficulties were included. However, we have no record of this and were not able to investigate whether these children show a differential impact of a SEE + .

To minimize testing burden on school, we evaluated children’s performance using bespoke booklets completed as a whole class rather than the established one-to-one administration by an experimenter. Although the five booklets were developed based on existing literature [86,87,90], a limitation of this approach is that it is difficult to directly compare the results of this study to the existing literature on the development of socio-emotional abilities. Another limitation of this study is that the socio-emotional measures were only collected after the intervention. However, the large sample, and blind within-school randomization of intervention conditions ensures that no systematic biases are present [67].

While expected age effects were observed in the emotion recognition and ToM task [91], ceiling effects for Year 5 children in the real photograph condition may have affected our ability to detect improvements from the intervention. Reversely, while in the vignette task (which did not show benefits from SEE+) accuracy was around 80% on the two cognitive ToM items, accuracy was around chance, ~ 40%, on the four affective ToM items, and developmental difference in performance on this task between Year 3 and Year 5 children was much smaller than in the emotion recognition and ToM task. The vignette task used for the present study had many fewer items than the original version used with adolescents and adults, which had ten items for each condition (cognitive ToM, affective ToM, physical causality [90]), and it is therefore possible that the affective ToM items were too difficult for the primary school children tested here.

### Future work

While we found beneficial effects of the SEE+ computerized intervention using cartoon characters, there was an issue of transfer of recognition of emotions from cartoon to real faces. Future work could assess whether greater transfer would be observed if real-world film images were used during the intervention. It would also be worth exploring in more detail the separate impacts of the scenarios as presented through the SEE+ computerized learning activity, and the whole class discussions around those topics mediated by the teacher to arrive at a classroom consensus response.

This work has focussed on 7- to 10-year-olds, but it could equally be extended to other age groups. However, age-appropriate curriculum items and assessments would need to be developed. Developing a program for pre-schoolers may help children acquire socio-emotional skills that would ready them to join primary school [97]. A previous intervention engaged preschoolers into discussions of storybook character’s mental states and found the intervention benefited false belief understanding but not emotion understanding or social competence [98]. The current approach, which includes a focus on character’s facial and body expression of emotions and complex social interactions, could complement such an intervention and lead to broader socio-emotional skills benefits. It may also be interesting to extend this to adolescence [16,17], which has been considered to be a sensitive period for sociocultural processing [99]. Adolescents experience greater independence and more complex social interactions than primary school children and evidence from social neuroscience indicates that adolescents’ brains undergo prolonged maturation supporting the integration of self and others’ perspectives [100]. Resources of the kind evaluated here may help them to better manage the challenges they face. The advantage of a computerized learning activity is that it can also be delivered online and at the user’s discretion, which may appeal more to adolescents.

As mentioned, the United Nations Educational, Scientific and Cultural Organization (UNESCO) is increasingly seeing socio-emotional cognition as a foundational cornerstone of learning and education across global contexts [9]. Future work could try to tailor the training to children with greater need and within special education schools in England and internationally. If successful, SEE+ training would provide an extremely easy to use and cost-effective educational and remedial tool.

## Conclusion

In summary, we have developed and assessed a computer-based whole class learning activity (SEE+) designed to improve emotion recognition and ToM skills in middle childhood. A large-scale randomised control trial revealed that children’s socio-emotional cognitive skills improved significantly following the SEE+ intervention. The benefits of the programme showed near transfer in both 7- to 8-year-olds and 9- to 10-year-olds. While further work will be needed to assess whether adapted stimuli can lead to far transfer of benefits, this study identifies a cheap and easy to use platform for delivering socio-emotional training across diverse educational contexts.

## Supporting information

S1 File Appendix. Design of SEE+ computerized classroom-based learning activities.
(DOCX)

S1 Fig Example of facial expression blending for complex emotions expressed by a SEE + virtual character. (DOCX)
