# Supplementary material for: SEE+ computerized classroom-based training enhances 7- to 10-year-olds' socio-emotional cognition through observation and inference
Source: PLoS One. 2025 Sep 2;20(9):e0330934. doi: 10.1371/journal.pone.0330934 (PMC12404391; doi:10.1371/journal.pone.0330934)
Supplement: S1 File — Design of SEE+ computerized classroom-based learning activities. (DOCX) [file pone.0330934.s001.docx]

# **S1 File. Appendix.** Design of SEE+ Computerized Classroom-based Learning Activities

## SEE+ Virtual characters animation

The virtual characters were two girls (Ella and Beth), and two boys (Andy and Dean), aged 6–10 years old. Andy, Dean and Ella were of White and Beth of Black African ethnicity and, Ella was the youngest character. Each of the four characters was endowed with a rich set of facial expressions (see **Fig S1**) and gestures, with minimal use of paralinguistic utterances such as ‘hello’, ‘thank you’, ‘Um’ and ‘Ahh’. The facial expressions were compounds of emotions composed from Ekman’s six basic emotional states [1], with valence from positive, through neutral, to negative. The range of gestures available to the characters included extending arms (up, down, sideways and diagonally); precise hand gestures, including extending digits while folding the rest of the fingers (necessary for precise pointing); and head movements (up, down, sideways and a combination thereof), which in combination with hand gestures, help disambiguate functions (e.g., specific objects the individual characters’ may point to).

SEE+ virtual characters’ social and emotional behaviours within a social scenario were scripted as sequenced chains according to four protagonist roles: (i) the first protagonist was an instigator who experienced an emotion that led to an action towards the second protagonist, which initiated a social dilemma or conflict between the four characters by inducing: (ii) a reactive passive or aggressive emotional response from the second protagonist (respondent), (iii) a counteraction response from the third protagonist (follower), who had been observing and reinforced the instigator’s response by affirming or assisting in the instigator’s actions, and (iv) a counter-reactive response from the fourth protagonist (bystander), who had also been observing, who intervened if and when needed to pro-socially resolve the social dilemma or conflict. The SEE+ cognitive architecture endows greater repertoire for face, eye and body gestures to Andy and Dean followed by Ella and Beth. Andy mostly plays roles of instigator or respondent, Dean plays all roles, Ella mostly plays as respondent or follower but sometimes instigator and, Beth, as bystander but sometimes also a follower.

The social scenario scripts were rendered as animations and embedded within the digital learning environment. Although SEE+ characters are underpinned with AI cognitive architecture, called FAtiMA [2], which typically drives the autonomous behaviours of these agents, in this version of the software all scenarios and characters’ behaviours were hand-animated to achieve the level of sophistication in the social interactions between autonomous agents required. The reason for hand-animating the scenarios is mainly technical, since coordinating autonomous behaviours involving multiple agents is considered one of the hardest problems in AI planning.

In this way the vicarious learning part of the SEE+ (i.e. the first and last phases of the interaction with the system, see below) was facilitated through animated recordings of social interactions between virtual characters to depict complex social emotions such as intimidation, ostracism, embarrassment, envy, empathy, guilt, shame, and joy.

## S1 Fig 1. Example of facial expression blending for complex emotions expressed by a SEE+ virtual character.

##
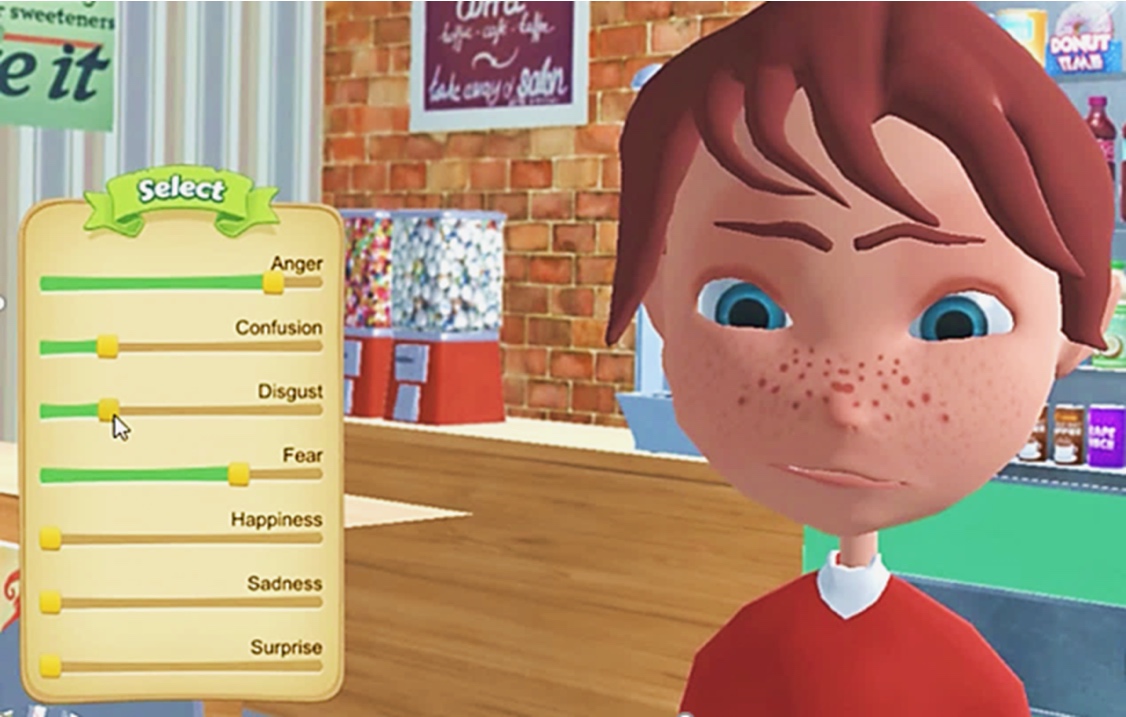


## The image shows the emotionally complex facial expressions available to create SEE+ characters’ emotions. The facial expression blending was developed during a related project called SHARE-IT, on which SEE+ builds (see Porayska-Pomsta et al. [3]).

# **SEE+ computerized, classroom-based learning activities**

The SEE+ computerized classroom-based learning activities were designed to train children’s socio-emotional cognition by: (i) *observing* animations of four virtual characters in social scenarios involving social dilemmas or conflict (implicit inference of characters’ mental states), and (ii) *inferring* what the characters were doing and feeling through reflective questioning (explicit reasoning about the characters’ mental states). Thirty learning activities were developed. Within the classroom, teachers projected the learning activities on interactive whiteboards and facilitated children in progressing through the different phases of the task by operating the computer interface.

### Phases of the social scenarios

The SEE+ whole class computerized learning activities were each structured into four phases of training presented as two episodes of implicit observational learning interleaved by explicit reflective questioning. Feedback was provided by a narrator’s voice-over recording with text guiding the learner through the phases. In *Phase 1*, children were provided with some context by text and narrative voice-over (main text Fig 1A) and then watched a first scripted animation of the four SEE+ characters interacting in a social scenario up to a cliff-hanger given the social dilemma or conflict (Fig 1B), followed by the presentation of three reflective questions, each with three possible answers, testing the children’s comprehension of what the characters were doing (Fig 1C). Feedback was provided by a tick indicating the correct answer.

In *Phase 2,* excerpts of the animation were replayed and children were asked two reflective questions about either the instigator or the respondent’s emotion and intensity of emotion as a consequence of the social dilemma or conflict (Figs 1E-F). The questions could be presented in one of six different formats: (i) selection from a choice of statements (Fig 1E), (ii) use of a slider to provide a rating of valence between two emotions (happiness and sadness in Fig 1F), (iii) use of a slider to provide a rating of the intensity of a single emotion, which when used would change the facial expression of the character on screen, and selection from a choice of images of a character’s (iv) whole face, (v) eyes, or (vi) gestures indicative of emotions. Feedback was provided using the character’s voice-over and corresponding text.

In *Phase 3*, children were asked to predict how either the instigator or the respondent might resolve the dilemma or conflict, using a single question with two possible choices of outcomes as a resolution (Fig 1G). Finally, in *Phase 4*, children watched a second scripted animation as feedback to show how the characters resolved the situation they found themselves in through prosocial behaviours, followed by further feedback from either the instigator or respondent’s voice-over with text.

In Fig 1, the embedded SEE+ learning activity archetype is perspective taking and inferring others’ intentions based on the sandbox false belief task [4] and the social scenario theme is ‘Teasing or Bullying?’. Here the context is that the characters are in the garden for an Easter egg treasure hunt. Andy (instigator), Dean (respondent) and Ella are findings eggs hidden in the garden and putting them in their basket while Beth is watching and setting up prizes, a large Easter egg and a trophy, for whoever finds the most eggs. Dean finds the most eggs and wins. Ella doesn’t find any eggs. Andy becomes unhappy when he doesn’t win, picks up Dean’s large Easter egg prize and hides it behind a tree while no one is looking. When Dean and Ella return from play, Dean becomes upset, Ella ignores Dean by looking away, Andy begins laughing at Dean. Ella then begins to laugh, and Beth is calm while she continues to watch. The social conflict is resolved when Andy runs behind the tree to pick up the large Easter egg and hands it back to Dean. The scenario ends with everyone laughing.

In this way the implicit observational learning episode engaged children in inferring the characters’ multiple mental states and the explicit reflective questions with feedback emphasised reflection on two-levels of mentalising: (i) the child can infer (i) that Andy is unhappy about not winning and (ii) that Andy thinks that Dean will expect to find his prize where Dean left it but Dean won’t know Andy hid his prize [5–8].

We included three scenarios addressing two sensitive topics about socio-cultural diversity in beliefs about religious celebrations and peer pressure to try smoking and drinking; topics that are addressed in the PSHE curriculum. Scenarios were age-specific for the younger age range for ethical reasons because depicting dilemmas or conflict between older children risked potential discomfort for younger children.

# **References**

1. Ekman P. An argument for basic emotions. Cogn Emot. 1992 May;6(3–4):169–200. <https://doi.org/10.1080/02699939208411068>

2. Bernardini S, Porayska-Pomsta K, Smith TJ, Avramides K. Building Autonomous Social Partners for Autistic Children. In: Nakano Y, Neff M, Paiva A, Walker M, editors. Intelligent Virtual Agents [Internet]. Berlin, Heidelberg: Springer Berlin Heidelberg; 2012 [cited 2024 May 24]. p. 46–52. (Hutchison D, Kanade T, Kittler J, Kleinberg JM, Mattern F, Mitchell JC, et al., editors. Lecture Notes in Computer Science; vol. 7502). <https://link.springer.com/10.1007/978-3-642-33197-8_5>

3. Porayska-Pomsta K, Anderson K, Bernardini S, Guldberg K, Smith T, Kossivaki L, et al. Building an Intelligent, Authorable Serious Game for Autistic Children and Their Carers. In: Reidsma D, Katayose H, Nijholt A, editors. Advances in Computer Entertainment [Internet]. Cham: Springer International Publishing; 2013 [cited 2024 May 25]. p. 456–75. (Lecture Notes in Computer Science; vol. 8253). <https://link.springer.com/10.1007/978-3-319-03161-3_34>

4. Sommerville JA, Bernstein DM, Meltzoff AN. Measuring Beliefs in Centimeters: Private Knowledge Biases Preschoolers’ and Adults’ Representation of Others’ Beliefs. Child Dev. 2013 Nov;84(6):1846–54. <https://doi.org/10.1111/cdev.12110>

5. Miller SA. Children’s understanding of second-order mental states. Psychol Bull. 2009;135(5):749–73. <https://doi.org/10.1037/a0016854>

6. O’Grady C, Kliesch C, Smith K, Scott-Phillips TC. The ease and extent of recursive mindreading, across implicit and explicit tasks. Evol Hum Behav. 2015 Jul;36(4):313–22. <https://doi.org/10.1016/j.evolhumbehav.2015.01.004>

7. Osterhaus C, Koerber S, Sodian B. Scaling of Advanced Theory‐of‐Mind Tasks. Child Dev. 2016 Nov;87(6):1971–91. <https://doi.org/10.1111/cdev.12566>

8. Wilson R, Hruby A, Perez-Zapata D, Van Der Kleij SW, Apperly IA. Is recursive “mindreading” really an exception to limitations on recursive thinking? J Exp Psychol Gen. 2023 May;152(5):1454–68. <https://doi.org/10.1037/xge0001322>
