# Supplementary figures and images for: SEE+ computerized classroom-based training enhances 7- to 10-year-olds' socio-emotional cognition through observation and inference
Source: PLoS One. 2025 Sep 2;20(9):e0330934. doi: 10.1371/journal.pone.0330934 (PMC12404391; doi:10.1371/journal.pone.0330934)

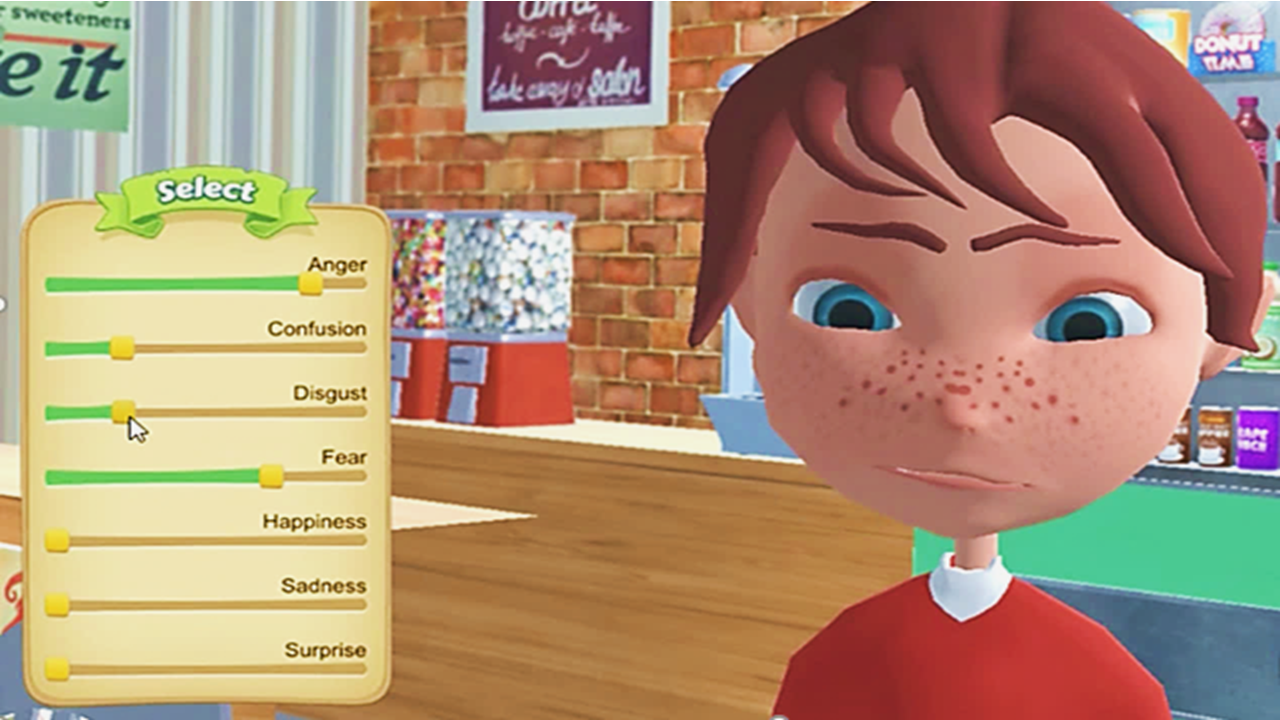

Supplement: S1 Fig — (DOCX) [file pone.0330934.s002.tif]
